# Multimorbidity of mental health and substance use disorders among housed and homeless U.S. veterans

**DOI:** 10.1038/s41598-025-99394-x

**Published:** 2025-04-30

**Authors:** Jack Tsai, Dorota Szymkowiak, Hind Beydoun

**Affiliations:** 1 United States Department of Veterans Affairs, Homeless Programs Office, Washington, DC USA; 2 Department of Management, Policy, and Community Health, University of Texas Health Science Center at Houston, 1200 Pressler St., Houston, TX 77030 USA

**Keywords:** Epidemiology, Mental disorders, Substance use disorders, Homeless persons, Psychology, Health care, Psychiatric disorders

## Abstract

Housing can be an important social determinant of mental health, which is why it is important to understand how mental health disorders (MHDs) and substance use disorders (SUDs) cluster and vary by housing instability. This study examined rates of comorbid MHDs and SUDs among three groups of U.S. veterans: those experiencing homelessness, those in supported housing, and those independently housed. Administrative data on a national cohort of 5,402,062 veterans (including 181,131 homeless veterans and 29,166 veterans in supported housing) enrolled in the U.S. Department of Veterans Affairs (VA) healthcare system from 2021 to 2023 were analyzed. Among homeless veterans, 28.3% had a SUD diagnosis, 62.9% a MHD diagnosis, and 24.7% comorbid MHD/SUD. Among veterans in supported housing, 38.2% had a SUD diagnosis, 67.3% a MHD diagnosis, and 32.0% comorbid MHD/SUD diagnoses. Among independently housed veterans, 7.9% had a SUD diagnosis, 41.6% a MHD diagnosis, and 5.8% comorbid MHD/SUD diagnoses. Homeless veterans with comorbid MHD/SUD used significantly more outpatient, inpatient, and emergency department care than veterans in supported housing; in turn, supported housing veterans with comorbid MHD/SUD used significantly more outpatient, inpatient, and emergency department care than independently housed veterans. Among homeless veterans with comorbid MHD/SUD diagnoses, those with addiction to central nervous system (CNS) stimulants used more outpatient, inpatient, and emergency department care than those with addiction to CNS depressants. In conclusion, the psychiatric epidemiology and comorbidity of veterans varies by housing situation, and there is continued need for psychiatric treatment among homeless veterans who enter supported housing.

**Supplementary Information:**

The online version contains supplementary material available at 10.1038/s41598-025-99394-x.

## Introduction

Many studies have reported that mental health disorders (MHDs) and substance use disorders (SUDs) are highly prevalent among homeless and unstably housed populations. MHD and SUDs have been found to be predictive of poorer housing and clinical outcomes among adults^1,2^, as well as being predictive of negative exits and returns to homelessness after obtaining a permanent housing situation^3,4^. However, most epidemiological studies on the topic in the United States (U.S.) have relied on small or single-site samples, or are outdated now^5–7^ because of two major challenges given the transitory and unstable situation surrounding homelessness. The first challenge is that homeless individuals are hard to recruit for studies because they may be living in places not meant for human habitation or may be temporarily staying in shelters. The second challenge is that many homeless individuals face competing demands for their health and social needs, so many may not be amenable to formal structured diagnostic interviews.

Because of these challenges, there is little understanding of the different types of MHDs and SUDs among homeless populations. For example, SUDs encompass addiction to a broad array of different types of substances and different SUDs may be differentially predictive of outcomes. For example, a few studies indicate homeless adults with stimulant use disorders or “uppers” (e.g., amphetamines, cocaine) may have greater difficulties with housing stability than those who have disorders related to central nervous system depressants or “downers,” like alcohol and benzodiazepines^8–10^. Greater understanding of specific disorders may inform development and deployment of appropriate interventions. Depending on the type of SUD, reviews of studies have reported that homeless adults can benefit from supervised consumption facilities, pharmaceutical interventions, or managed alcohol programs^11^. Moreover, there are dual disorders programs that are effective for unstably housed adults with comorbid MHDs and SUDs^12–14^.

One notable study using data from Wave 3 of the National Epidemiological Survey of Alcohol and Related Conditions (NESARC-III), which were collected between 2012 and 2013 based on structured interviews and diagnostic criteria in the Diagnostic and Statistical Manual of Mental Disorders, Fifth Edition (DSM-5)^15^, found that rates of Axis I MHDs ranged from 6 to 26% among adults with past-year experiences with homelessness with the most common being major depressive disorder followed by posttraumatic stress disorder and generalized anxiety disorder; substance use disorders (excluding tobacco) ranged from 0 to 37% with the most common disorders being alcohol, cannabis, and opioid use disorders^2^. However, these estimates were based on a sample collected over a decade ago and there is evidence that the psychiatric epidemiology of homeless adults has not remained static. One study that examined three different samples of homeless adults in St. Louis, Missouri in 1980, 1990, and 2000 reported that the prevalence of mood and substance use disorders among homeless adults has dramatically increased^16^. In particular, the largest increases were found in drug use disorders, particularly cocaine, and among women. Other studies, such as a recent systematic review, have found that cocaine use is prevalent in many homeless samples, with a median prevalence of lifetime cocaine use disorder of 30% across studies^17^. A more contemporary epidemiological study is needed as an update to these older studies.

Veterans are a unique subgroup in U.S. society and the U.S. Department of Veterans Affairs (VA) operates the largest healthcare and homeless service system in the country, serving over 100,000 homeless veterans annually^18^. The VA’s comprehensive medical record system provides a unique opportunity to analyze data on MHDs and SUDs in large samples of homeless adults. A study conducted over a decade ago reported on the prevalence of alcohol and drug use disorders among a national sample of veterans entering the Housing and Urban Development-Veterans Affairs Supportive Housing (HUD-VASH) program^19^. The HUD-VASH program provides rental subsidies as HUD Sect. 8 vouchers and case management services through the VA, and exists in all 50 states. Eligibility for the program are veterans who are experiencing homelessness, meet the criterion of low-income (i.e., 80% of the Area Median Income), and have a need for case management as determined by VA^20^.

The study reported 60% of veterans in the supportive housing program had SUDs, and 54% of those with SUDs had both alcohol and drug use disorders. Veterans in the HUD-VASH program with both alcohol and drug use disorders had more extensive homeless histories and had more recent use of transitional housing or residential treatment than those with either alcohol or drug use disorder alone. However, further expansive work is needed to fill gaps in current knowledge of the prevalence of different types of SUDs in broader samples of homeless veterans, the extent of comorbidity between MHDs and SUDs, and understanding how MHDs and SUDs are related to different types of healthcare use.

The current study aims to provide an in-depth epidemiological examination of major MHD and SUD diagnoses in a contemporary, large cohort of homeless veterans in the VA healthcare system. We seek to identify not only common single MHD/SUD diagnoses in isolation, but common clustering of comorbid MHD/SUD diagnoses. To examine differences between important veteran subgroups, we compared homeless veterans, veterans in HUD-VASH, and veterans who were independently housed.

## Methods

National VA data were extracted from two major sources: the VA’s Corporate Data Warehouse (CDW) and the VA’s Homeless Operations Management and Evaluation System (HOMES). CDW contains medical records information and was used to obtain data on diagnoses and health services use among veterans. HOMES contains data on workload and services provided in VA homeless programs entered by VA homeless staff and was used to obtain information on use of VA homeless services.

In this study, homelessness was defined using VA’s operational definition^21^, which included three indicators: International Classification of Diseases, Tenth Revision, Clinical Modification (ICD-10-CM) code Z59.0; use of any VA Homeless Programs (excluding HUD-VASH and the Veterans Justice Programs)^22^, and/or screening positive on VA’s homelessness screening clinical reminder^23^. We also included veterans who self-reported literal homelessness at the time of their intake into VA homeless programs as recorded in HOMES or upon entry into the Supportive Services for Veteran Families (SSVF) program. Homeless veterans who met one or more of these criteria were grouped together in the first group. Veterans who moved into a HUD-VASH unit within the study period, as recorded in HOMES, were grouped into a second group. The third group consisted of independently housed veterans, which were defined as veterans who screened negative on VA’s homeless screening clinical reminder.

Using a study period from July 1, 2021, to June 30, 2023, we extracted data on a cohort of 5,402,062 veterans (including 181,131 homeless veterans; 29,166 HUD-VASH veterans; and 5,191,765 independently housed veterans). For each subgroup, the index date was the veteran’s earliest indication of group membership, within the study period. We extracted ICD-10-CM codes in the veteran’s inpatient and outpatient records for one year following the index date. Clinical diagnoses of MHDs and SUDs were based on these ICD-10 codes, organized into categories catalogued with published definitions on the VA intranet in the ALEX platform^24^ by the VA’s Program Evaluation and Resource Center (PERC). The main diagnostic categories and corresponding codes used in this study are listed in the Appendix.

### Data analysis

Analyses proceed in four phases. First, descriptive statistics and chi-square tests were used to describe and compare sociodemographic, military, and clinical diagnoses among homeless, HUD-VASH, and independently housed veterans. Second, we identified the most prevalent combinations of MHD and SUD diagnoses using several methods, including identifying pairs of diagnoses and comorbid MHD and SUD diagnoses. Third, generalized linear models (GLM) were constructed to compare homeless, HUD-VASH, and independently housed veterans with comorbid MHD and SUD diagnoses on differences in healthcare utilization, controlling for all sociodemographic characteristics. Unstandardized coefficients with 95% confidence intervals were generated. Finally, we used the same GLM approach to examine differences in healthcare utilization between homeless veterans with SUD diagnoses based on diagnoses related to central nervous system (CNS) stimulants versus CNS depressants. CNS simulants were categorized as cocaine and amphetamine, while CNS depressants were categorized as alcohol, opioids, cannabis, and sedatives. Three sets of GLM analyses were conducted on the sample of homeless veterans; the first set compared homeless veterans with only SUD on addiction to CNS stimulants versus CNS depressants, the second set compared homeless veterans with comorbid MHD and SUD on addiction to CNS simulants versus CNS depressants; and the third set compared homeless veterans with severe MHD (categorized as schizophrenia-spectrum disorder, other psychosis, bipolar disorder) and comorbid SUD on addiction to CNS stimulants versus CNS depressants. Veterans with both CNS stimulant and depressant use disorders were excluded from this third set of analyses. All analyses were performed using SAS Enterprise Guide, version 8.3. Due to the retrospective nature of the study, the requirement for obtaining informed consent was waived and study procedures were approved by the VA’s institutional review board.

## Results

Table 1 shows the prevalence rates of different SUDs and MHDs among veterans experiencing homelessness, veterans enrolled in the HUD-VASH program, and veterans who were independently housed. Among the three groups, the highest prevalence rates of different SUD diagnoses were found among HUD-VASH veterans followed by homeless veterans and then independently housed veterans. Rates of MHDs, on the other hand, were similar among HUD-VASH and homeless veterans, with independently housed veterans consistently having much lower rates across MHD diagnoses. Among homeless veterans (*n* = 181,131), 28.3% had a SUD diagnosis and 62.9% had a MHD diagnosis. For specific SUD diagnoses, 19.4% had diagnosis of alcohol use disorder and 19.6% had diagnosis of drug use disorder; 24.7% had comorbid MHD and SUD diagnoses. Among HUD-VASH veterans (*n* = 29,166), 38.2% had a SUD diagnosis and 67.3% had a MHD diagnosis; 24.8% had diagnosis of alcohol use disorder and 27.7% had diagnosis of a drug use disorder; and 32.0% had comorbid MHD and SUD diagnoses. Among independently housed veterans (*n* = 5,191,765), 7.9% had a SUD diagnosis and 41.6% had a MHD diagnosis; 5.8% had diagnosis of alcohol use disorder and 3.2% had diagnosis of drug use disorders; and 5.8% had a comorbid MHD and SUD diagnosis.

**Table 1:** Comparison of sociodemographic and clinical characteristics of veterans who are independently housed, in the HUD-VASH program, and who are currently homeless.

Variable	Housed (*N* = 5,191,765)		HUD-VASH (*N* = 29,166)		Homeless (*N* = 181,131)		Chi-square test^1^	Δ% between independently housed and HUD-VASH	Δ% between HUD-VASH and homeless
	*N*	%	*N*	%	*N*	%			
Age							59084.85		
17–39	770,494	14.8%	4,760	16.3%	47,180	26.0%		−1.5%	−9.7%
40–49	581,634	11.2%	3,648	12.5%	26,831	14.8%		−1.3%	−2.3%
50–59	771,904	14.9%	5,885	20.2%	32,000	17.7%		−5.3%	2.5%
60–69	971,495	18.7%	11,213	38.4%	45,383	25.1%		**−19.7%**	**13.4%**
70–79	1,515,245	29.2%	3,134	10.7%	24,488	13.5%		**18.4%**	−2.8%
80+	580,792	11.2%	523	1.8%	5,233	2.9%		9.4%	−1.1%
Race/ethnicity							49223.80		
Non−Hispanic white	3,355,383	64.6%	12,711	43.6%	82,401	45.5%		**21.0%**	−1.9%
Non−Hispanic black	873,371	16.8%	10,821	37.1%	62,081	34.3%		**−20.3%**	2.8%
Hispanic	394,818	7.6%	2,078	7.1%	14,248	7.9%		0.5%	−0.7%
Mixed race/other	187,271	3.6%	1,340	4.6%	7,116	3.9%		−1.0%	0.7%
Missing	380,721	7.3%	2,213	7.6%	15,269	8.4%		−0.3%	−0.8%
Sex							2150.69		
Male	4,633,421	89.2%	25,400	87.1%	155,597	85.9%		2.2%	1.2%
Female	558,143	10.8%	3,763	12.9%	25,518	14.1%		−2.2%	−1.2%
Marital status							100167.43		
Married	2,966,424	57.1%	4,024	13.8%	47,189	26.1%		**43.3%**	**−12.3%**
Single/never married	760,451	14.6%	9,554	32.8%	51,558	28.5%		**−18.1%**	4.3%
Divorced/separated	1,168,473	22.5%	14,108	48.4%	73,346	40.5%		**−25.9%**	7.9%
Widowed	197,412	3.8%	1,022	3.5%	5,342	2.9%		0.3%	0.6%
Missing	98,804	1.9%	455	1.6%	3,680	2.0%		0.3%	−0.5%
Percent service−connected disability							8232.58		
None/0%	1,553,210	29.9%	14,067	48.2%	62,041	34.3%		**−18.3%**	**14.0%**
10–40%	915,565	17.6%	6,260	21.5%	26,372	14.6%		−−−−3.8%	6.9%
50–100%	2,722,789	52.4%	8,836	30.3%	92,702	51.2%		**22.1%**	**−20.9%**
Combat exposure	593,598	11.4%	1,854	6.4%	19,758	10.9%	782.70	5.1%	−4.6%
Military sexual trauma	287,214	5.5%	3,619	12.4%	21,622	11.9%	15535.31	−6.9%	0.5%
Diagnoses									
Any substance use disorder (SUD)	412,273	7.9%	11,136	38.2%	51,283	28.3%	122268.52	**−30.2%**	9.9%
Alcohol use disorder	303,097	5.8%	7,242	24.8%	35,131	19.4%	70401.63	**−19.0%**	5.4%
Any drug use disorder	163,722	3.2%	8,088	27.7%	35,417	19.6%	173037.64	**−24.6%**	8.2%
Opioid use disorder	36,598	0.7%	1,788	6.1%	8,462	4.7%	41500.44	−5.4%	1.5%
Cannabis use disorder	103,386	2.0%	3,977	13.6%	20,336	11.2%	80835.20	**−11.6%**	2.4%
Sedative use disorder	6,013	0.1%	240	0.8%	1,873	1.0%	10708.21	−0.7%	−0.2%
Cocaine use disorder	20,207	0.4%	3,029	10.4%	11,906	6.6%	146580.59	**−10.0%**	3.8%
Amphetamine use disorder	13,880	0.3%	2,590	8.9%	10,187	5.6%	144303.80	−8.6%	3.3%
Hallucinogen use disorder	1,132	0.0%	103	0.4%	595	0.3%	5743.95	−0.3%	0.0%
Inhalant use disorder	248	0.0%	17	0.1%	144	0.1%	1390.51	−0.1%	0.0%
Other drug use disorder	18,776	0.4%	1,870	6.4%	9,454	5.2%	92689.68	−6.0%	1.2%
Any mental health disorder (MHD)	2,161,789	41.6%	19,622	67.3%	113,850	62.9%	39624.87	**−25.6%**	4.4%
Any mood disorder	1,212,254	23.3%	13,537	46.4%	79,954	44.1%	49200.13	**−23.1%**	2.3%
Major depression	839,963	16.2%	9,223	31.6%	56,488	31.2%	32892.00	**−15.4%**	0.4%
Bipolar disorder	109,882	2.1%	2,535	8.7%	14,313	7.9%	30723.21	−6.6%	0.8%
Other mood disorder	527,467	10.2%	5,919	20.3%	36,760	20.3%	21997.00	**−10.1%**	0.0%
Any psychosis	55,726	1.1%	1,743	6.0%	9,466	5.2%	30048.60	−4.9%	0.8%
Schizophrenia-spectrum disorder	53,614	1.0%	1,676	5.7%	9,219	5.1%	29572.93	−4.7%	0.7%
Other psychosis	2,750	0.1%	104	0.4%	489	0.3%	1743.63	−0.3%	0.1%
Any anxiety disorder	871,273	16.8%	8,219	28.2%	50,052	27.6%	16948.31	**−11.4%**	0.5%
Generalized anxiety disorder	254,802	4.9%	2,551	8.7%	15,425	8.5%	5590.75	−3.8%	0.2%
Other anxiety-related disorder	733,485	14.1%	6,876	23.6%	42,830	23.6%	14741.44	−9.4%	−0.1%
Any stress-related disorder	1,246,309	24.0%	11,349	38.9%	72,284	39.9%	27080.17	**−14.9%**	−1.0%
Posttraumatic stress disorder	1,021,624	19.7%	8,108	27.8%	57,252	31.6%	16577.47	−8.1%	−3.8%
Non-PTSD stress disorder	347,723	6.7%	5,057	17.3%	26,854	14.8%	22473.74	**−10.6%**	2.5%
Other mental health disorder	390,949	7.5%	4,079	14.0%	26,188	14.5%	13250.49	−6.5%	−0.5%
Comorbid MHD and SUD	298,819	5.8%	9,328	32.0%	44,805	24.7%	134380.94	**−26.2%**	7.2%

^1^ All Chi-square tests were significant at the *p* < .001 level.

Homelessness was defined based on an established operational definition^21^. Supported housing was based on enrollment in the Housing and Urban Development-Veterans Affairs Supportive Housing (HUD-VASH) program. Housing stability was based on a negative screen on the Homelessness Screening Clinical Reminder. Bolded values indicate ≥∆±10%.

Among notable differences (≥∆10%) in sociodemographic and clinical characteristics between HUD-VASH veterans and homeless veterans, HUD-VASH veterans were older (i.e., more likely to be 60 or over) and more likely to be unmarried and to have none/0% VA service-connected disability. Compared to independently housed veterans, HUD-VASH veterans were younger (i.e., less likely to be 70 or over); more likely to be non-Hispanic black, unmarried, and to have none/0% VA service-connected disability. HUD-VASH veterans were also more likely to have alcohol and drug use disorders; mood, anxiety, and stress disorders; and comorbid MHD and SUD diagnoses than independently housed veterans. While not specifically shown in Table 1, as expected, there were many large differences (≥∆10%) between independently housed and homeless veterans on clinical diagnoses, with homeless veterans having higher rates of MHD and SUD diagnoses, and comorbid MHD and SUD diagnoses (24.7% versus 5.8%).

Veterans who were homeless, in HUD-VASH, or independently housed differed not only on the prevalence of different MHD and SUD diagnoses and their rate of comorbidities, but also showed some differences in their most common clustering of diagnoses. As shown in Table 2, major depression with other MHD, and PTSD with other MHD were among the most common pairs of diagnoses across all three groups. The most common comorbid MHD and SUD differed between groups; with alcohol use disorder and PTSD being the most common among independently housed veterans, and drug use disorder and other MHD being the most common among both HUD-VASH and homeless veterans.

**Table 2:** Most common Multimorbidity among mental health disorders (MHDs) and substance use disorders (SUDs) in veterans who are independently housed, in the HUD-VASH program, or are currently homeless.

Independently housed (*N* = 5,145,197)			
Most common pair of diagnoses	N	% of total sample	% of those with any MHD or SUD (*N* = 2,275,328)
1. Posttraumatic stress disorder (PTSD & other MHD	404,098	7.8%	17.8%
2. Major depression & PTSD	396,802	7.6%	17.4%
3. Major depression & other MHD	390,495	7.5%	17.2%
Most common comorbid MHD & SUD			
Alcohol use disorder & PTSD	122,544	2.4%	5.4%
Alcohol use disorder & other MHD	112,701	2.2%	5.0%
Alcohol use disorder & major depression	106,260	2.0%	4.7%
**HUD-VASH (** ***N*** ** = 29, 166)**			
Most common pair of diagnoses	N	% of total sample	% of those with any MHD or SUD (*N* = 21,433)
Major depression & other MHD	5,175	17.7%	24.1%
PTSD & other MHD	4,565	15.7%	21.3%
Drug use disorder & other MHD	4,419	15.2%	20.6%
Most common comorbid MHD & SUD			
Drug use disorder & other MHD	4,419	15.2%	20.6%
Alcohol use disorder & drug use disorder	4,194	14.4%	19.6%
Alcohol use disorder & other MHD	3,856	13.2%	18.0%
**Homeless (** ***N*** ** = 181,131)**			
Most common pair of diagnoses	N	% of total sample	% of those with any MHD or SUD (*N* = 120,336)
Major depression & other MHD	33,809	18.7%	28.1%
PTSD & other MHD	32,053	17.7%	26.6%
Major depression & PTSD	30,307	16.7%	25.2%
Most common comorbid MHD & SUD			
Drug use disorder & other MHD	20,887	11.5%	17.4%
Alcohol use disorder & other MHD	20,156	11.1%	16.7%
Alcohol use disorder & drug use disorder	19,267	10.6%	16.0%

MHD = Mental Health Disorders; SUD = Substance Use Disorder.

To examine how homeless, HUD-VASH, and independently housed veterans with dual diagnoses differed on healthcare use, multivariable analyses were conducted to compare the three groups with any comorbid MHD/SUD diagnoses. As shown in Table 3, homeless veterans consistently had more outpatient mental health, substance abuse treatment, primary care, rehabilitation, and ancillary/diagnostic visits; more emergency department visits; and more mental health, substance abuse, medical, and long-term care inpatient admissions and longer total inpatient stays than both HUD-VASH veterans and independently housed veterans. Compared to independently housed veterans, HUD-VASH veterans had more outpatient mental health, substance abuse treatment, rehabilitation, and ancillary/diagnostic visits; more emergency department visits; and more inpatient admissions and longer total inpatient stays across all categories.

**Table 3:** Multivariable analysis comparing homeless veterans, HUD-VASH veterans, and independent housed veterans with any comorbid mental health and substance use disorders on past year service utilization.

Variable	HUD-VASH veterans vs. independently housed veterans	Homeless veterans vs. HUD-VASH veterans	Homeless veterans vs. independently housed veterans
Outpatient visits			
Mental health	10.04 (9.70-10.39)***	7.66 (6.74–8.59)***	18.49 (18.28–18.70)***
Substance abuse	7.93 (7.65–8.22)***	5.65 (4.83–6.47)***	14.67 (14.48–14.85)***
Primary care	−0.33 (−0.45–0.21)***	0.96 (0.78–1.14)***	0.70 (0.64–0.76)***
Medical specialty	−0.76 (−0.96–0.56)***	0.57 (0.34–0.79)***	−0.10 (−0.20–0.00)*
Rehabilitation	1.02 (0.80–1.23)***	2.46 (2.12–2.80)***	3.49 (3.38–3.60)***
Ancillary/diagnostic	2.07 (1.77–2.37)***	3.08 (2.65–3.50)***	5.36 (5.21–5.51)***
Emergency department visits	0.68 (0.64–0.72)***	0.26 (0.18–0.34)***	0.93 (0.91–0.95)***
Inpatient admissions			
Mental health	0.18 (0.17–0.19)***	0.11 (0.09–0.13)***	0.30 (0.29–0.30)***
Substance abuse	0.05 (0.05–0.05)***	0.03 (0.02–0.03)***	0.08 (0.07–0.08)***
Medical/surgical	0.12 (0.11–0.14)***	0.09 (0.06–0.12)***	0.21 (0.21–0.22)***
Long-term care	0.03 (0.03–0.04)***	0.05 (0.04–0.06)***	0.08 (0.08–0.09)***
Total inpatient nights			
Mental health	1.85 (1.71–2.00)***	3.47 (2.83–4.11)***	5.47 (5.34–5.59)***
Substance abuse	1.87 (1.78–1.97)***	1.04 (0.68–1.40)***	2.84 (2.77–2.91)***
Medical/surgical	0.62 (0.47–0.77)***	1.19 (0.86–1.52)***	1.67 (1.59–1.75)***
Long-term care	1.55 (1.15–1.95)***	4.01 (3.20–4.82)***	5.57 (5.34–5.79)***

Multivariable analyses controlled for all sociodemographic characteristics. All groups shown had comorbid mental health and substance use disorders. Values shown are unstandardized coefficients with 95% confidence intervals.

Lastly, Table 4 shows results of three separate analyses on only the homeless veteran sample, comparing healthcare use among homeless veterans with different types of SUD diagnoses grouped with MHD diagnoses. In the first set of analyses among homeless veterans with only a SUD diagnosis, those with addiction to a CNS stimulant (cocaine, amphetamine) were compared to those with addiction to a CNS depressant (alcohol, opioids, cannabis, sedatives). The results showed that those with addiction to a CNS stimulant had slightly fewer outpatient substance abuse treatment and primary care visits, but more emergency department visits.

**Table 4:** Multivariable analysis comparing only homeless veterans with different groupings of mental health disorder (MHDs) and substance use disorder (SUD) diagnoses with focus on central nervous system (CNS) stimulants versus depressants.

Variable	Veterans with SUD (all single diagnoses only) CNS stimulant vs. CNS depressant disorders	Veterans with SUD and comorbid MHD (all dual diagnoses) CNS stimulant vs. CNS depressant disorders	Veterans with SUD and severe MHD (all dual diagnoses) CNS stimulant vs. CNS depressant disorders
Outpatient visits			
Mental health	0.10 (−0.71–0.91)	−4.92 (−6.31–3.52)***	−9.93 (−13.01–6.85)***
Substance abuse	−1.01 (−2.02–0.01)*	−4.52 (−5.73–3.31)***	−6.02 (−8.22–3.81)***
Primary care	−0.90 (−1.44–0.37)***	−0.99 (−1.31–0.67)***	−1.43 (−2.06–0.80)***
Medical specialty	0.29 (−0.47–1.04)	−0.32 (−0.72–0.08)	−1.09 (−1.73–0.45)***
Rehabilitation	−0.43 (−1.13–0.27)	−0.50 (−1.05–0.05)	−1.99 (−3.07–0.91)***
Ancillary/diagnostic	0.34 (−0.76–1.45)	−0.64 (−1.34–0.05)	−3.15 (−4.43–1.86)***
Emergency department visits	0.29 (0.12–0.47)**	0.24 (0.12–0.37)***	0.07 (−0.26–0.41)
Inpatient admissions			
Mental health	0.00 (−0.01–0.01)	−0.01 (−0.04–0.01)	−0.10 (−0.17–0.03)**
Substance abuse	0.00 (−0.00–0.01)	−0.01 (−0.02–0.00)*	−0.01 (−0.03–0.01)
Medical/surgical	0.12 (0.05–0.20)**	−0.03 (−0.07–0.02)	−0.13 (−0.22–0.04)**
Long-term care	0.01 (−0.01–0.02)	−0.01 (−0.02–0.00)	−0.03 (−0.05–0.01)**
Total inpatient nights			
Mental health	0.09 (−0.64–0.82)	−0.44 (−1.48–0.61)	−2.67 (−4.81–0.53)*
Substance abuse	0.11 (−0.42–0.63)	−0.52 (−0.97–0.08)*	−0.49 (−1.32–0.34)
Medical/surgical	0.07 (−0.74–0.89)	0.53 (−0.00–1.06)	−0.29 (−1.46–0.89)
Long-term care	−0.34 (−2.92–2.25)	−1.59 (−3.01–0.17)*	−3.90 (−6.89–0.91)*

Multivariable analyses controlled for all sociodemographic characteristics. CNS stimulants include cocaine, amphetamine. CNS depressants include alcohol, opioids, cannabis, and sedatives. Severe MHD is defined as schizophrenia-spectrum, other psychotic disorder, or bipolar disorder. Values shown are unstandardized coefficients with 95% confidence intervals.

In the second set of analyses among homeless veterans with comorbid MHD and SUD diagnoses, those with an addiction to a CNS stimulant were compared to those with an addiction to a CNS depressant. These results showed that those with an addiction to a CNS simulant had fewer outpatient visits across all types; more emergency department visits; fewer inpatient admission across types; and shorter total inpatient stays for mental health, substance abuse treatment, and long-term care.

In the third set of analyses among homeless veterans with a severe MHD (schizophrenia-spectrum disorder, other psychotic disorder, bipolar disorder) and comorbid SUD, those with an addiction to a CNS stimulant were compared to those with an addiction to a CNS depressant. The results showed that compared to homeless veterans with an addiction to a CNS depressant, those with an addiction to a CNS simulant had fewer outpatient visits across all types; fewer inpatient admissions for all but substance abuse treatment; and shorter total inpatient stays in mental health and long-term care.

## Discussion

This large national study provides epidemiological descriptive data on the diagnosis rates of different MHDs and SUDs among homeless, supported housing, and independently housed veterans. There were clear differences in rates of many MHD and SUD diagnoses with higher rates consistently seen among homeless veterans and veterans in the HUD-VASH program compared to independently housed veterans across diagnoses. This is generally consistent with previous studies^7,19^, although our contemporary rates of SUD were lower than those reported in older studies of homeless adults^16,17^ and veterans in HUD-VASH^19^ using varying methodologies. Our study contributes to the literature by providing specific current prevalence rates across individual diagnoses documented in medical records.

What was somewhat surprising was our finding that there were higher rates of SUD diagnoses among HUD-VASH veterans than homeless veterans. Rates of comorbid MHD/SUD diagnoses were also highest among HUD-VASH veterans affecting about one-third (32%) followed by a quarter (25%) among homeless veterans and about one-twentieth (6%) among independently housed veterans. There may be several possible explanations for this. First, since the HUD-VASH program has implemented the Housing First model which seeks to provide immediate access to housing with no prerequisites for sobriety or participation in treatment^25,26^, obstacles to accessing supported housing for veterans with SUD have been removed which may have allowed for greater enrollment. Second, veterans in HUD-VASH may be more likely to have accurate diagnoses because they are more stably housed and connected to healthcare than homeless veterans. And finally, it is possible that some veterans in HUD-VASH experienced a substance abuse relapse after entering supported housing, as a higher risk for drug overdose deaths have been found among HUD-VASH veterans compared to homeless veterans over certain periods of time in supported housing^27^.

Importantly, there were also large differences in MHD and SUD diagnoses between the two groups of housed veterans, i.e., those in HUD-VASH and those independently housed. Veterans in HUD-VASH were more likely to have a SUD diagnosis than independently housed veterans, particularly for a drug use disorder (30% higher proportion). HUD-VASH veterans were also more likely to have a MHD diagnosis than independently housed veterans, including a 15% higher proportion with major depression and a 26% higher proportion with comorbid MHD and SUD diagnoses. These findings indicate that many veterans in supported housing continue to experience major mental health and substance use problems. These findings may also reflect the program’s Housing First approach and suggest the importance of wrap-around mental health and substance abuse treatment in supported housing.

Across the three groups with any MHD or SUD, 17–28% had major depression with some other MHD. Some of the most common pairs of MHD/SUD diagnoses or comorbid MHD and SUD diagnoses were similar across the three groups. However, there were certain pairs/comorbid diagnoses that were particularly common in certain groups. For example, alcohol use disorder appeared to be more common as a comorbid disorder among independently housed veterans, while drug use disorder was more common as a comorbid disorder among homeless and HUD-VASH veterans. These findings suggest there are some similar patterns among veterans in general, but also the heterogeneity of clinical needs among veterans in different states of housing and homelessness. Further research is needed to pinpoint if and how these different patterns of MHDs and SUDs contribute to or are the consequence of homelessness through longitudinal studies to examine any differential outcomes.

A deeper analysis of homeless veterans with different types of addictive disorders revealed some interesting findings. Homeless veterans with addiction to CNS stimulants generally tended to use less outpatient health care services, but were more frequent users of emergency department services than those with addiction to CNS depressants. These findings extend those of a few previous studies that have reported stimulant users tend to have worse housing outcomes in supported housing than others^8,10^, thus special attention and outreach may be needed to connect those addicted to CNS stimulants with substance abuse treatment. The exception was among homeless veterans with severe MHDs and addictive disorders, which found no difference between those addicted to CNS simulants versus depressants on use of emergency department services. Perhaps symptoms of severe MHDs take precedence over any differences between CNS simulants and depressants. These findings need to be replicated with other samples before stronger conclusions can be made.

This study had several limitations worth noting. First, the study was based on VA administrative data which relied on the accurate and timely documentation of clinicians. Prevalence rates of MHDs and SUDs reported were based on diagnoses recorded in medical records, and may not reflect the true prevalence rate in overall representative populations. Second, homeless veterans are heterogeneous and may have been receiving various VA homeless services but were grouped together in this study. Third, we only analyzed pairs of diagnoses and comorbid MHDs and SUDs, but multimorbidity with more than two psychiatric diagnoses and including other medical diagnoses is not uncommon among homeless populations^28–30^. Fourth, it is unknown how generalizable these findings are to veterans not enrolled in VA healthcare or other homeless adults who are not veterans, so further study with other samples is needed.

These limitations notwithstanding, this study examines the rates of MHDs and SUDs of nearly 200,000 homeless veterans and 30,000 veterans in supported housing. Comorbid MHDs and SUDs were examined in detail by diagnosis type, and healthcare use by addiction to CNS stimulants and depressants. The findings describe the continued psychiatric challenges experienced by veterans who enter supported housing. Together, the study findings provide a comprehensive snapshot of MHDs and SUDs among veterans in different stages of housing and homelessness, which can inform policies and practices to meet their healthcare needs.

## Electronic supplementary material

Below is the link to the electronic supplementary material.

Supplementary Material 1

## Data Availability

De-identified data is available upon request from the corresponding author with proper approval from institutional review boards and the VA Homeless Programs Office. Programming code used for analysis is available upon request.
