# Supplementary material for: Multimorbidity of mental health and substance use disorders among housed and homeless U.S. veterans
Source: Sci Rep. 2025 Apr 30;15:15185. doi: 10.1038/s41598-025-99394-x (PMC12043799; doi:10.1038/s41598-025-99394-x)
Supplement: Supplementary file 1 — Supplementary Material 1 [file 41598_2025_99394_MOESM1_ESM.docx]

**Appendix.** ICD-10 codes used for diagnoses of mental health disorders and substance use disorders

| **Diagnoses** | **ICD-10 Codes** |
| --- | --- |
| Schizophrenia spectrum disorder | F20 and F25 |
| Other psychosis | F06.0, F06.1, F06.2, F22, F23, F24, F28, F29, F53.1 |
| Bipolar spectrum disorder | F30 (except F30.4) and F31 (except F31.72, F31.74, F31.76) |
| Major depression | F32.0, F32.1, F32.2, F32.3, F32.9, F33.0, F33.1, F33.2, F33.3, F33.9 |
| Other mood disorder | F06.3, F32.8, F32.A, F33.8, F34, F39, and F53.0 |
| Generalized anxiety disorder | F41.1 |
| Anxiety related disorder | F06.4, F40, F41 (except F41.1), F42, F45.2 (except F45.22) |
| Posttraumatic stress disorder | F43.1 |
| Non-PTSD stress disorder | F43 (except F43.1), F94.1, and F94.2 |
| Alcohol use disorder | F10 (except F10.11, F10.21, and F10.91) |
| Other mental health disorder | All other codes (not already captured above) for F21, F44-F45, F48, F50-F52, F54-F55, F59-F60, F63-F66, F68-F69, F90-F91, F93-F95, F98-F99 |
| Opioid use disorder | F11 (except F11.11, F11.21, and F11.91) |
| Cannabis use disorder | F12 (except F12.11, F12.21, and F12.91) |
| Sedative use disorder | F13 (except F13.11, F13.21, and F13.91) |
| Cocaine use disorder | F14 (except F14.11, F14.21, and F14.91) |
| Amphetamine use disorder | F15 (except F15.11, F15.21, and F15.91) |
| Hallucinogen use disorder | F16 (except F16.11, F16.21, and F16.91) |
| Inhalant use disorder | F18 (except F18.11, F18.21, and F18.91) |
| Other psychoactive drug use disorder | F19 (except F19.11, F19.21, and F19.91) |

:,
